# C/EBPβ cooperates with MYB to maintain the oncogenic program of AML cells

**DOI:** 10.18632/oncotarget.28377

**Published:** 2023-03-11

**Authors:** Karl-Heinz Klempnauer

**Keywords:** MYB, C/EBPbeta, p300, GFI1, AML

## Abstract

Studies on the role of transcription factor MYB in acute myeloid leukemia (AML) have identified MYB as a key regulator of a transcriptional program for self-renewal of AML cells. Recent work summarized here has now highlighted the CCAAT-box/enhancer binding protein beta (C/EBPβ) as an essential factor and potential therapeutic target that cooperates with MYB and coactivator p300 in the maintenance of the leukemic cells.

## INTRODUCTION

The *MYB* gene initially attracted attention as the progenitor of a retrovirally-transduced oncogene that induces a myeloid leukemia in chicken [1–4]. *MYB* encodes a transcription factor with essential roles in the development of the hematopoietic system [5] and the proliferation and differentiation of hematopoietic progenitor cells [6]. Subsequent work identified MYB also as a crucial player in the development and maintenance of leukemia in humans. Recurrent genomic rearrangements of the *MYB* gene resulting in increased expression were detected in T-cell acute lymphoblastic leukemia (T-ALL) [7–9]. Recently, mutations that generate de novo MYB binding sites in the vicinity of the oncogenes *TAL1* and *LMO2* were found to create MYB-dependent super enhancers that drive the aberrant expression of these genes in T-ALL [10, 11]. In acute myeloid leukemia (AML), recurrent changes of *MYB* appear to be rare, rather, *MYB* is thought to act downstream of the causative events in the maintenance of the leukemic cells [12–14]. As a result, AML cells are dependent on high levels of MYB expression, making them more sensitive than normal hematopoietic progenitor cells to strategies of down-modulation of its expression [15, 16]. This was elegantly shown in a mouse model of AML, where decreased MYB expression eradicated the leukemia without abolishing normal hematopoiesis [14]. These findings suggested partial inhibition of MYB as a therapeutic strategy for AML and led to first attempts to identify pharmacological inhibitors of MYB. Incidentally, interest in MYB as a drug target is not restricted to hematopoietic malignancies. For example, *MYB* also plays a prominent role in the development of adenoid cystic carcinoma (ACC), where recurrent translocations between *MYB* and *NFIB* generate aberrant MYB/NFIB fusion proteins [17, 18].

### The MYB-p300 interaction: the “achilles heel” of MYB

Attempts to devise inhibitors of MYB so far mainly focused on the disruption of the cooperation between MYB and the coactivator p300. Transcriptional stimulation of target genes by MYB strongly depends on its association and cooperation with p300 which binds via its so-called KIX domain, located in the N-terminal half of the coactivator, to a LXXLL amino acid sequence motif in the MYB transactivation domain [19]. That p300 is a crucial cooperation partner of MYB in hematopoietic cells was demonstrated by mutations in the LXXLL motif or KIX domain, which decrease MYB activity by weakening the interaction of both proteins and cause various hematopoietic defects in mice [20, 21]. Furthermore, Pattabiraman et al., showed that the interaction of MYB and p300 is required for the induction of acute myeloid leukemia by human AML oncogenes [22]. Consequently, the disruption of the interaction of MYB and p300 appeared to be a suitable strategy for targeting MYB. Studies from our and other laboratories showed that disruption of the MYB-p300 interaction, mediated either by low molecular weight compounds or by a peptide mimetic, inhibits the proliferation of leukemic cells. Importantly, leukemic cells from AML patients or from mouse AML models were inhibited significantly stronger than normal hematopoietic progenitor cells, consistent with the addiction of the leukemic cells to high MYB levels. Moreover, these studies also revealed a delay of the development of AML in mouse models *in vivo* [23–27]. In addition to the disruption of the MYB-p300 interaction there are also promising reports showing that the expression of MYB, rather than its activity, is amenable to targeting by pharmacologic agents [28, 29]. One of these agents, mebendazole, an approved drug for the treatment of worm infections, is particularly interesting as it can be re-purposed to downregulate MYB expression by inducing degradation of the protein.

### Recent studies implicate C/EBPβ in the maintenance of AML cells

Recent work also highlighted the transcription factor CCAAT-box/enhancer binding protein beta (C/EBPβ) as a relevant factor and potential drug target in AML [30]. C/EBPβ is a conserved member of basic-region-leucine-zipper (bzip) family of DNA binding proteins that serves a broad spectrum of functions in fundamental cellular processes, including proliferation and differentiation of different cell types [31, 32]. Recent studies showed that C/EBPβ interacts with a variety of proteins, presumably reflecting the complexity of its functions [33]. A link between MYB and C/EBPβ was initially established by showing that C/EBPβ co-operates with MYB in myeloid-specific gene expression in chicken cells. As an example, MYB and C/EBPβ both bind to the enhancer and the promoter of *MIM-1*, a direct MYB target gene specifically expressed in myeloid cells, and co-operatively drive the transcription of the gene [34–37]. The synergy between MYB and C/EBPβ was strongly stimulated by the direct interaction of both protein with the transcriptional coactivator p300 [38]. Together, this implicated MYB, C/EBPβ and p300 in a transcriptional module that drives myeloid-specific gene expression.

Recent work suggested that this module also plays a role in human AML cells. Roe et al., studied the genome-wide chromatin occupancy of specific transcription factors in human AML cells and found that MYB, C/EBPβ and p300, as well as several other proteins, are associated with the promoter and enhancer regions of multiple genes in these cells, consistent with a role of these proteins in gene expression in myeloid cells [39]. Our own work showed that the natural compound helenalin acetate as well as several related compounds that were initially identified as potent MYB inhibitors using a *MIM-1* based screening system [40, 41], upon further characterization turned out to inhibit the activity of C/EBPβ rather than that of MYB [42]. Interestingly, although these compounds failed to inhibit MYB directly, transcriptional profiling showed that they largely mimicked the effect of MYB knock-down in AML cells [43, 44]. AML cells treated with helenalin acetate or related C/EBPβ inhibitory compounds ceased to proliferate and succumbed to differentiation or cell death. These anti-proliferative effects were significantly diminished upon ectopic expression of C/EBPβ or of MYB, supporting the notion that these compounds disrupt the activity of the MYB- and C/EBPβ-dependent transcriptional module. Finally, primary AML cells from mice or from human AML patients were more sensitive to the anti-proliferative effects of the compounds than normal murine or human hematopoietic stem cells [43]. Overall, these data confirmed the close link between MYB and C/EBPβ in AML cells and provided strong evidence that C/EBPβ, as part of a transcriptional MYB-C/EBPβ-p300 module, is required to maintain the viability of AML cells. Independent support for a role of C/EBPβ in maintaining the proliferative state of AML cells also came from recent work on *MLL-ENL* transformed AML cells, which showed that C/EBPβ is required for the maintenance of these leukemic cells [45].

A role of C/EBPβ in the maintenance of AML cells was surprising considering previous studies that identified C/EBPβ as a differentiation-promoting factor in all-trans retinoic acid (ATRA) treated acute promyelocytic leukemia (APL) cells [46]. This appeared to contradict a pro-leukemogenic role of C/EBPβ and suggested that C/EBPβ may have opposing functions in AML cells. How can this be rationalized? It is conceivable that the expression level of C/EBPβ is responsible for switching between these opposing functions because C/EBPβ expression is strongly increased in ATRA-treated cells and may override its pro-leukemic function observed at lower levels of C/EBPβ. However, since we observed that increased ectopic expression of C/EBPβ alone was unable to induce differentiation in AML or APL cells [43], it appears more likely that ATRA treatment affects the expression of additional factors switching C/EBPβ from a pro-leukemogenic to a differentiation-inducing protein. Intriguingly, MYB itself might play a role in this switch because ATRA was recently found to decrease MYB expression due to long-range effects mediated by distant *MYB* enhancer elements [47]. In this hypothetic scenario, the level of MYB and the ability to form the MYB-C/EBPβ-p300 module may allow C/EBPβ to switch between its alternative pro-leukemic and differentiation-inducing functions.

### GFI1 is a crucial downstream target of the transcriptional MYB-C/EBPβ-p300 module

The idea that AML cells are critically dependent on the activity of a combinatorial MYB-C/EBPβ-p300 transcriptional module would gain credibility if a relevant downstream target controlled by this module could be identified. Transcriptional profiling of AML cells treated with or without helenalin acetate or related natural compounds pinpointed the growth factor independent 1 (*GFI1*) gene as one of the genes whose expression was downregulated by the compounds. The downregulation occurred on a short time course, arguing against indirect effects induced by the compounds [43]. *GFI1* encodes a zinc finger DNA-binding protein that is part of repressive GFI1/CoREST chromatin complexes. In myeloid cells, these complexes are known to suppress the expression of differentiation-associated genes, hence, it was concluded that GFI1 is required for the maintenance of hematopoietic stem and progenitor cells [45–50]. We therefore suspected that the expression of *GFI1*, which was already known as a MYB target [51], might be controlled by MYB in combination with C/EBPβ and p300. This turned out to be true. Firstly, inspection of published chromatin occupancy data [39] showed that MYB, C/EBPβ and p300 co-occupied the promoter of the *GFI1* gene in human AML cells. Secondly, reporter studies demonstrated that the *GFI1* promoter was synergistically activated by all three proteins, thus identifying *GFI1* as a *bona fide* target of the MYB-C/EBPβ-p300 transcriptional module [43]. Importantly, ectopic expression of GFI1 rescued AML cells from the anti-proliferative effect of helenalin acetate and prevented their differentiation, whereas overexpression of an inactive mutant of GFI1 had no significant effect [43]. This demonstrated that the anti-proliferative activity of the C/EBPβ-inhibitory compounds was mediated the downregulation of GFI1. Overall, these studies pinpoint GFI1 as a relevant downstream gene and support the notion that C/EBPβ as part of a MYB-, C/EBPβ- and p300-dependent transcriptional module plays an essential role in the maintenance of the leukemic cells. This makes C/EBPβ an interesting pro-oncogenic player in AML, worthy of further studies.
